# On the Search for Potentially Anomalous Traces of Cosmic Ray Particles in Images Acquired by Cmos Detectors for a Continuous Stream of Emerging Observational Data

**DOI:** 10.3390/s24061835

**Published:** 2024-03-13

**Authors:** Marcin Piekarczyk, Tomasz Hachaj

**Affiliations:** Faculty of Electrical Engineering, Automatics, Computer Science and Biomedical Engineering, AGH University of Krakow, Al. Mickiewicza 30, 30-059 Krakow, Poland; thachaj@agh.edu.pl

**Keywords:** high-energy particles, image-based detection, anomalies detectionl, principal components analysis, image processing, sequential karhunen-loeve transform, big data, citizen science

## Abstract

In this paper we propose the method for detecting potential anomalous cosmic ray particle tracks in big data image dataset acquired by Complementary Metal-Oxide-Semiconductors (CMOS). Those sensors are part of scientific infrastructure of Cosmic Ray Extremely Distributed Observatory (CREDO). The use of Incremental PCA (Principal Components Analysis) allowed approximation of loadings which might be updated at runtime. Incremental PCA with Sequential Karhunen-Loeve Transform results with almost identical embedding as basic PCA. Depending on image preprocessing method the weighted distance between coordinate frame and its approximation was at the level from 0.01 to 0.02 radian for batches with size of 10,000 images. This significantly reduces the necessary calculations in terms of memory complexity so that our method can be used for big data. The use of intuitive parameters of the potential anomalies detection algorithm based on object density in embedding space makes our method intuitive to use. The sets of anomalies returned by our proposed algorithm do not contain any typical morphologies of particle tracks shapes. Thus, one can conclude that our proposed method effectively filter-off typical (in terms of analysis of variance) shapes of particle tracks by searching for those that can be treated as significantly different from the others in the dataset. We also proposed method that can be used to find similar objects, which gives it the potential, for example, to be used in minimal distance-based classification and CREDO image database querying. The proposed algorithm was tested on more than half a million (570,000+) images that contains various morphologies of cosmic particle tracks. To our knowledge, this is the first study of this kind based on data collected using a distributed network of CMOS sensors embedded in the cell phones of participants collaborating within the citizen science paradigm.

## 1. Introduction

The problem of automatic anomaly detection is seen as one of the significant challenges in the analysis and recognition of measurement data. Anomaly detection concerns the search for those observations that deviate from the definition of normality for the considered set of observations [1,2]. Sometimes, interchangeable terms such as outlier detection or novelty detection are also used in this context, although they are not necessarily completely analogous [3,4,5,6,7]. This area has been actively developed in recent years, and many methods have been proposed in this field of research [8,9]. Among the first techniques proposed to deal with anomalies detection were statistical methods [10,11], especially those related to density estimation like KDE (Kernel Density Estimation) [12]. Nowadays, many solutions apply various machine learning methods, like shallow and deep models [5,13,14].

Anomaly detection techniques are applied to analyze and solve a wide range of problems in various areas. Examples of practical applications include cybersecurity (intrusion detection systems) [15,16,17,18,19], economy and healthcare (fraud detection) [20,21,22,23,24,25], industry (fault diagnosis, damage detection) [26,27,28,29,30,31], medicine (medical diagnosis, disease outbreak detection) [32,33,34,35,36], earth sciences (event detection) [37,38,39,40], bioinformatics [36,41,42,43], genetics [44,45], physics [46,47,48,49,50] or astronomy [51,52,53,54,55,56].

The ability to detect non-trivial observations that deviate from a consistent data stream is a particularly big challenge in particle physics and astronomy [57]. The search for unusual data can lead to the discovery of unknown physical phenomena [58,59,60]. Creating effective tools for such type of automatic analysis and identification is a very important research subject in particle physics and astronomy.

Research in particle physics is performed on data acquired from experiments performed on large-scale stationary particle accelerators at projects such as LHC/CERN (Large Hadron Collider) [61,62,63,64], SLAC (Stanford Linear Accelerator Center) [65], Thomas Jefferson National Accelerator Facility [66,67], J-PARC (Japan Proton Accelerator Research Complex) [68,69] and many others. There are also large-scale observatories that measure cosmic radiation arriving from space. Among them are Pierre Auger Observatory [70,71], IceCube [72] or Telescope Array Project [73]. Stationary observatories of this type perform very accurate measurements. However, the observations they make are limited to the area where their research infrastructure is located. Due to this fact, they observe only a certain fraction of cosmic radiation reaching the Earth’s atmosphere. To overcome the limitations of stationary observatories, several projects have been developed in recent years that allow distributed observations of cosmic radiation. These projects are based on the citizen science paradigm and use CMOS/CCD camera-based particle detectors [74]. Projects of this kind are:CRAYFIS (Cosmic RAYs Found In Smartphones) [75,76], DECO (Distributed Electronic Cosmic-ray Observatory) [77,78] and CREDO [79,80]. CRAYFIS [81] is a globally distributed network of cosmic-ray sensors for the exploration of cosmic rays, with the potential to reveal unexpected or previously unobserved planet-scale phenomena such as widely separated simultaneous extensive air showers. DECO is a similar project which utilizes smartphone-based cosmic rays detectors. The project is conducting advanced research on detection and classification of particle types based on deep learning models [82]. CREDO project uses smartphone-based detectors and additionally integrates data from other sources such as simple scintillation detectors [83,84,85,86,87]. The data collected by the CREDO project is stored in open repositories and are available for scientific purposes. In all of these projects, the use of mobile detectors based on optical sensors that record traces of particle radiation energy offers great flexibility and the possibility to extend observation coverage on a global scale [81]. Detectors acquires a huge amount of measurement data of various types, which requires appropriate analysis especially automatic recognition in big data streams [88,89].

The main purpose of searching for unusual signals (anomalies) in such data sets is to look for new physical phenomena (unknown physics) [79]. Such new phenomena might be potentially registered as non-typical particle traces observed on detector arrays and might be evidence of new particles or physical interactions. Such phenomena can occur when ultra high-energy cosmic radiation strikes the Earth and creates a stream of secondary particles observed by detectors. It should be noted that the primary particles hitting the atmosphere can have energies far beyond the energy ranges achievable in Earth’s laboratories, creating unique physical conditions. Detection of unusual particle images observed on a globally distributed set of detectors also has the potential to reveal unexpected or previously unobserved phenomena occurring at the planetary scale [81]. Such phenomena might be revealed for example if similar types of anomalies occur in remote geographic locations corresponding to independent or simultaneous extensive air showers (EAS). Statistical analysis of anomalies in a large dataset is also a useful tool for tuning detection and filtering algorithms for observed events. Such analysis also makes it possible to study the response of a variety of CMOS sensors to radiation by analysis of a statistically significant number of actual measurements.

The problem addressed in this paper concerns the detection of anomalies in CREDO data acquired from smartphone-based mobile detectors. The primary carrier of information in this case are images of particle tracks recorded on CMOS arrays [74,90]. Since the data is collected in continuous mode, it is necessary to take into account the possibility of streaming digging through the dataset for unusual observations. So far CREDO data has been analyzed for both background signal filtering and artifacts removal [91,92,93], as well as classification and recognition [92,94,95,96]. There have also been initial works on detecting abnormal data based on various techniques such as rough sets [97].

### 1.1. Novelty of This Research

To our knowledge, this is the first study which proposes a method that can detect potential anomalies in a continuous data stream and find objects with similar morphological structure in cosmic rays unlabeled data collected by a distributed network of CMOS sensors embedded in the cell phones. The proposed solution has been implemented and validated on the largest dataset of its kind to date, containing over 570,000 images. An important fact is that our approach has no limitations due to the size of the dataset, as embedding can be calculated and updated relatively quickly using small batches of new data. We were able to achieve this by using incremental PCA (Principal Components Analysis) feature extractions [98,99,100,101], appropriate image preprocessing and density-based anomalies search. In practice, the method presented in this paper has the potential for immediate detection of potential anomalies in the data stream incoming from the entire CREDO observatory network.

### 1.2. Paper Structure

The rest of the article is organized as follows. Section 2 discusses the structure of the CREDO data subset used in the article, explains the preprocessing of the raw image data, the mathematical basis of Incremental PCA, and the scheme of the anomaly detection algorithm. We have divided the presentation and discussion of the results into two sections. Section 3 presents technical aspects of the proposed method. Section 4 contains detailed discussion and interpretation of results. Section 5 summarizes the scientific contributions.

## 2. Material and Methods

Since the research problem addressed in this article concerns the search for anomalies in CREDO imaging data, it is necessary to start by defining how we understand these anomalies. Due to the nature of the observations, we are dealing with traces left by energy-carrying particles on a CMOS array. From a physics point of view high-energy particles should left traces in the shape of low-density point or thin lines [80]. Observations of this shape are the majority of the dataset. Patterns that deviate significantly from these standards can be treated as anomalies. We are unable to identify a reference pattern for anomalies, as they can have morphologically very different shapes.

### 2.1. Datasets

The CREDO dataset is currently the largest open dataset containing recorded traces of potential cosmic ray particles acquired by mobile detectors. To our knowledge, there is no other such comprehensive dataset of this modality, which is additionally constantly updated with new recorded events. For this reason, we applied it to our research as a state-of-the-art data repository in the field of citizen science-based cosmic rays observations. A subset of CREDO data from Android-based mobile detectors was used to verify the solutions proposed in the article. It consists a set of observations saved in digital images with resolution 60×60. Each recorded observation is also associated with metadata such as acquisition time, geographic coordinates, etc. Those additional information is not considered in the algorithms presented. The data was recorded during 2023 year and passed the standard anti-artifact filter used in the project [74]. The size of the dataset we used in this research is 573,335 images.

### 2.2. Image Prepossessing (Aligning)

Image aligning might improve results of further image analysis [102,103,104,105]. In case of CREDO dataset, the aligning is based on translating images so that the pixels with the highest grayscale intensity will be in the center of the image, and rotating images so that the brightest collinear pixels will be horizontal. This type of alignment might be done with the aid of PCA. The proposed aligning algorithm works as follows:

- Input image is converted to grayscale;
- PCA is computed on a dataset constructed from pixels of grayscale image. Each pixel has its coordinate in the image. If the pixel is black (has value equals 0) its coordinates are not included in the dataset. If the pixel has a value greater than zero, we add to the dataset as many points with coordinates of that pixel as the value of that pixel (from 1 to 255). This means that the brighter the pixel is, the more data it appends to the dataset from which the PCA is calculated;
- Most significant PCA axis is used to rotate image while dataset mean is used to translate image;
- After image rotation and translation result image is cropped to original size of input image. Due to this fact some pixels in image borders might not have calculated pixels value. In order to calculate those border pixels we perform pixel extrapolation. We have used following pixel extrapolation methods which are defined in OpenCV [106] (see Table 1).
  - B. Constant–no matter of image colors “abcd”, border (not defined by transform) pixels are assigned to have constants color “o”.
  - B. Reflect–border pixels (not defined by transform) are reflections of image colors. For example if image colors are “abcd” left border will have extrapolated values “…dcb” and right border will have extrapolated values “cba…”.
  - B. Replicate–border pixels (not defined by transform) are the same pixels that are positioned on the edge of image which has pixels defined by a transform. For example if image colors are “abcd” left border will have extrapolated values “…aaa” and right border will have extrapolated values “ddd…”.

The images are converted to grayscale as part of processing, however in all figures we present original images in RGB color scale.

The proposed algorithm pseudo code is presented in Algorithm 1. In Table 1 we present image aligning methods we used during dataset preprocessing. We have tested four methods: no preprocessing (None) which use raw data and Algorithm 1 with all three extrapolation methods we have described above.

### 2.3. PCA-Based Features

Principal components analysis is a statistical method based on covariance analysis that finds the transformation matrix which allows projecting the dataset to lower dimension with linear transform that preserves maximal number of information in the sense of preserving variance. In other words object in a dataset can be described with fewer dimension than with initial one. There are several methods that can be used in place of PCA for feature extractions. Popular methods of this type include Independent component analysis (ICA) [107] or utilizing the latent space from various Encoder-Decoder deep neural networks (E-D) [108]. Among the most important limitations of PCA are the facts that it is only a linear transformation, as well as usually requires scaling of individual features. In our case, feature scaling is not necessary because we are dealing with image files where the signal representation is limited and quantized. Unlike ICA, PCA does not require that the signals we want to extract meet the assumptions of independence, having non-Gaussian histograms, and having lower complexity than mixture signals. PCA also has some advantages over the E-D approach. PCA allows exact calculation of information loss due to dimensionality reduction, since in PCA one can easily estimate the percentage of variance explained by a subset of the selected coordinate system axes. Thanks to that, one can control the size of PCA embedding without the necessity of recalculating the projection matrix. In case of E-D the latent space is derived from the network’s bottleneck and its size cannot be modified without retraining the whole architecture.
**Algorithm 1:** Image aligning PCA-based algorithm [109]
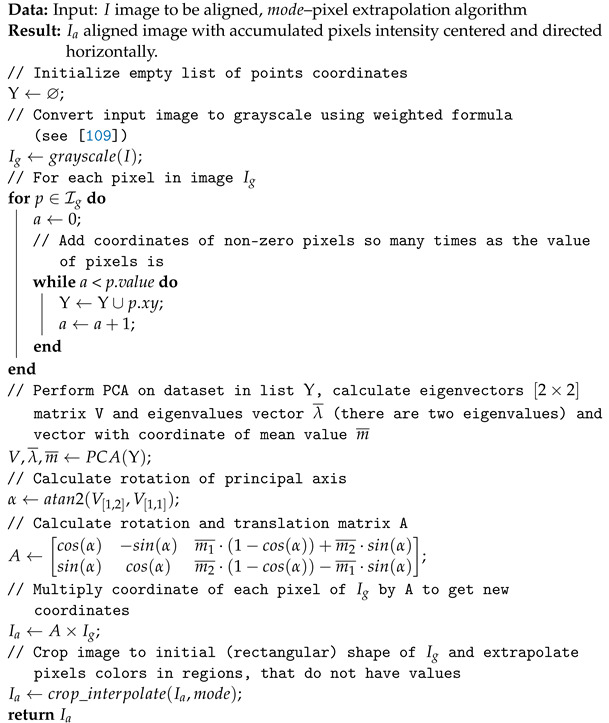


    Let the dataset Ψ contains *n* images. Each image $I_{i}$ has resolution $d_{1}\times d_{2}$, i∈[1…n]. Values of pixels in image are in range [0,1]. For the rest of the paper let us consider two-dimensional image as one dimensional vector of length $d=d_{1}\cdot d_{2}$. To make this 2d to 1d conversion we store each row of the image one by another in a single row vector. In order to calculate PCA-based embedding (we will call it also “basic PCA”) of image we can adapt eigenfaces-based image representation similar to one used in [110]. In order to do so we need to create a matrix that contains all observations:

(1) $$X\leftarrow ={\left[I_{1},\ldots ,I_{n}\right]}^{T}$$

where *X* has n×d dimensions. The number of rows is the same as the number of images in the dataset while number of columns is the same as images dimension. The next step is to calculate mean vector of each column of the matrix *X*, $mean_{col}\left(X\right)$ and then covariance matrix:

(2) $$T=\frac{{\left(X-mean_{col}\left(X\right)\right)}^{T}\times \left(X-mean_{col}\left(X\right)\right)}{d}$$

where *T* is a square d×d matrix.

Matrix *T* is then a subject of eigendecomposition in order to find set of eigenvectors stored in matrix *V* and corresponding to them eigenvalues $\vec{\lambda }$. Let us assume that PCA loadings are positioned in columns. After ordering eigenvectors in order of descending absolute values of eigenvalues the embedding *E* is calculated according to equation:

(3) $$E={\left(V\times {\left(X-mean_{col}\left(X\right)\right)}^{T}\right)}^{T}$$

where *E* is a n×d dimensional embedding. In order to perform dimensional reduction, we need to skip certain rows in matrix *V*, for example when we leave only 5 first rows the embedding will be 5 dimensional and matrix *E* will become n×5.

### 2.4. Potential Anomalies Detection

After applying PCA and dimensions reduction we can use a new obtained embedding (latent) space to examine similarity between objects. We can define an anomaly as an object that is not similar to other objects in the dataset in terms of distance between objects embedding. According to this definition outliers might be considered as anomalies. In order to detect outliers we can apply certain cluster analysis algorithm like agglomerative clustering [111], DBSCAN [112] or even *k*-means [97]. In case of first two algorithms in order to optimize performance it is required to calculate distance matrix between objects in the dataset which might be difficult or hardly possible in case of big data. In our case we do not need to find the answer to which cluster a certain objects belongs, rather if a certain object is outlier. Knowing this we can adapt the anomalies searching approach derived from the DBSCAN: an object $I_{j}$ with embedding $E_{j}$ is an outlier when in its neighbourhood with radius ϵ there are less than *k* other objects. The anomalies set *A* can be defined as:

(4) $$I_{j}\in A\Leftrightarrow \#\left\{I_{i}:d\left(E_{j},E_{i}\right)<\epsilon ,i\in \left[1\ldots n\right]\right\}<k$$

where # is cardinal number of the set and $\left\{I_{i}:d\left(E_{j},E_{i}\right)<\epsilon ,i\in \left[1\ldots n\right]\right\}$ is a set of objects which distances between their embedding and embedding of $I_{j}$ is less than ϵ and *d* is a distance function (in our case Euclidean distance).

The algorithm that detect potential anomalies according to Equation (4) has complexity $O\left(n\right)=n^{2}$ however because evaluation of each object $I_{j}$ in the dataset is independent of the others it can be easily speed up by the map-reduce approach on the parallel processing pipeline.

### 2.5. Querying the Object Database for the Most Similar Objects

The procedure of finding k most similar objects to $I_{j}$ requires calculating distance between embedding of this object and embedding of each other object and ordering them in descending order. Objects corresponding to first k smallest distances indicate most similar objects.

(5) $$D_{i}st\left(E_{j}\right)=\left[\left(d\left(E_{1},E_{j}\right),E_{1}\right),\ldots ,\left(d\left(E_{i},E_{j}\right),E_{i}\right),\ldots ,\left(d\left(E_{n},E_{j}\right),E_{n}\right)\right]\;ordered\;by\;d\left(E_{i},E_{j}\right)$$

where $D_{i}st\left(E_{j}\right)$ is ordered list of pairs, each pair contains distance between $E_{j}$ and certain element from the dataset Ψ. Pairs in list are ordered by descending order by calculated distance, i∈[1…n]/j.

Formally set of *k* most similar objects is defined as:

(6) $$Sim\left(I_{j},k\right)=\left\{I_{i}:position\left(E_{i}\right)\;in\;D_{ist}\left(E_{j}\right)⩽k\right\}$$

where $position(E_{i})$ returns index of element $E_{i}$ in ordered list $D_{ist}\left(E_{j}\right)$.

According to Equation (6) if two or more objects have the same distance to $E_{j}$ it is possible that more than *k* objects will be returned.

Searching the image database for the *k* most similar objects to $I_{j}$ thus reduces to finding all $I_{i}$ that satisfy (6). If Algorithm 1 for image aligning and (3) or Algorithm 2 for embedding calculation is applied, the search process reduces to the pairwise distance calculation problem.

### 2.6. Approximation of PCA for Big Data

The calculation of PCA features with an algorithm given in the Section 2.3 has a memory and computational bottleneck when the covariance matrix is calculated according to Equation (2). The rest of the computation is done on a fixed-size matrix. The matrix *X* (see (1)) occupies $n\cdot d_{1}\cdot d_{2}\cdot bc$ in memory, where bc is the number of bytes allocated to represent the floating-point number. For the real world data considered in this work, in the case of large image datasets, for example, with quantity of $10^{6}$ images and a resolution of 60×60 pixels, the matrix T stored with double precision (8 Bytes) occupies in memory: $10^{6}\cdot 60\cdot 60\cdot 8\approx 26.8$ GB and grows linearly as the number of images in the dataset increase. In order to reduce the memory and computational complexity of the algorithm finding image embedding, one can use PCA approximation based on incremental calculation of PCA with, for example, the algorithm proposed in the paper [101]. That algorithm is an extension of the Sequential Karhunen-Loeve Transform [113]. A mean update is calculated according to a Youngs and Cramer variance update procedure [114]. The method is called Incremental PCA and works as described in Algorithm 2. Returned matrices $V_{a}^{T}$ and $S_{a}$ being approximations of PCA can be used in (3) to calculate embedding.

### 2.7. Detecting Potential Anomalies in Big Dataset under Condition of Continuously Incoming Objects

To perform anomaly detection on the dataset described in Section 2.1, one needs to do image aligning using Algorithm 1, generate an embedding of the dataset using Equation (3) and then use (4) at a fixed (ϵ,k). However, this approach requires calculating the memory-expensive Equation (2). If cosmic ray particle images are acquired continuously, the Equation (2) will have to be repeated from time to time, for example, when a new large enough batch of data is collected. We cannot assume that the dataset we have gathered so far is representative and skip updating (3), because new devices may be incorporated into the CREDO sensor network, from which the resulting data will have different characteristic from those acquired earlier. This will also requires updating the statistical parameters obtained from the PCA. In order to reduce the number of necessary calculations, the step of determining PCA with a basic algorithm, for example, based on SVD (Singular value decomposition), can be replaced by approximation of PCA by Algorithm 2. Algorithm 2 will be run every time a batch of new data of the certain size is collected. The rest of the data processing pipeline will look identical like in the case with the basic PCA. Note that (3) can be performed iteratively for individual images or groups of images, not necessarily for all of *X* at once. Anomalies detection with (4) can be run after each update of embedding, either for whole *X* or only for new objects in batch. The procedure depends on the strategy adopted, for example, whether one wants to repeatedly analyze the same (old) data in search of potential anomalies. Evaluation of the dependence of the obtained embedding on the size of the training dataset and the differences in the found anomalies will be analyzed in the following sections.
**Algorithm 2:** Incremental PCA algorithm
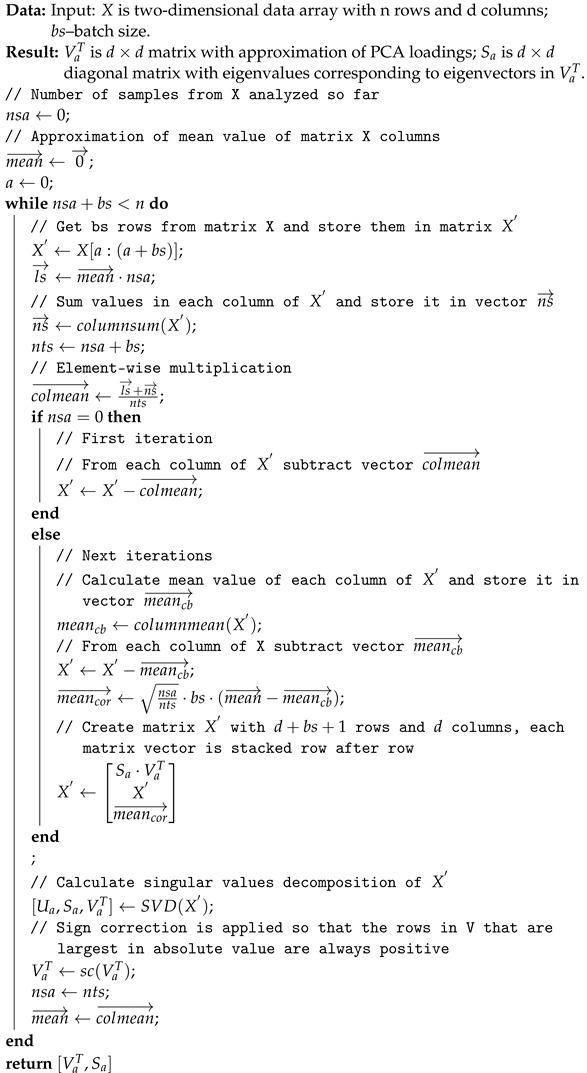


## 3. Results

We implemented our solution using Python 3.8. The source code of the proposed algorithm and the dataset can be downloaded from GitHub repository https://github.com/browarsoftware/anomalies_bigdata (accessed on 20 December 2023). We have used numba 0.5, numpy 1.22, opencv-python 4.5, scikit-learn 1.0, scipy 1.8 Python libraries. Plots were made in R langauge 3.6.

The purpose of the evaluation was to test the effectiveness of detecting potential anomalies using image preprocessing (aligning) methods described in Section 2.2, PCA-based features described in Section 2.3 and anomalies detection approach in Section 2.4.

The dataset presented in Section 2.1 was set randomly. In Table 2 we present a comparison of the resulting coordinate frames computed using basic PCA for four different preprocessing algorithms. The comparison of axes is intended to numerically calculate the difference between the potential embeddings and to indicate the effect of using different preprocessing methods on the calculation of PCA. The comparison of coordinate systems is done using coordinate frames weighted distance (cfd):

(7) $$cfd\left(\left(V_{1},\bar{\lambda_{1}}\right),\left(V_{2},\bar{\lambda_{2}}\right)\right)=\sum_{i=1}^{d}\frac{\bar{\lambda_{1,i}}+\bar{\lambda_{2,i}}}{2}∡\left(sc\left(V_{1,i}\right),sc\left(V_{2,i}\right)\right)$$

where $\left(V_{1},\bar{\lambda_{1}}\right)$ is eigenvectors matrix and eigenvalues vector of first PCA, $\left(V_{2},\bar{\lambda_{2}}\right)$ is eigenvectors matrix and eigenvalues vector of second PCA, $\left(V_{1,i},\lambda_{1,i}\right)$ is i-th eigenvector and *i*-th eigenvalue of first PCA, *sc* is a sign correction (see Algorithm 2) and *∡* is an operator for calculating the angle between vectors. Note that all eigenvalues of PCA are non-negative; cfd is measured in radians (rad).

To perform embedding we used 62 features out of 3600 that is, we reduced the dimensionality of embedding to 62 dimensions. Such a reduction explains, depending on the preprocessing method adopted, between 98% and 99% of the total variance in our dataset. We decided to adopt such a number of dimensions because it allowed us to more easily manipulate the value of ϵ in (4), which must be determined depending on the number of dimensions and in practice cannot be determined other way than experimentally, as in DBSCAN algorithm.

We made a comparison of the sets of potential anomalies returned by the method described by Equation (4) for different preprocessing algorithms from Section 2.2 and embedding calculated with basic PCA algorithm from Section 2.3. We used Jaccard index (*J*) [115] and Overlap coefficient (*OC*) [116] to compare the sets of anomalies:

(8) $$J\left(A_{1},A_{2}\right)=\frac{A_{1}\cap A_{2}}{A_{1}\cup A_{2}}$$

where $A_{1},A_{2}$ are potential anomalies sets to be compared.

(9) $$OC\left(A_{1},A_{2}\right)=\frac{A_{1}\cap A_{2}}{min(\#A_{1},\#A_{2})}$$

**Figure 1:**
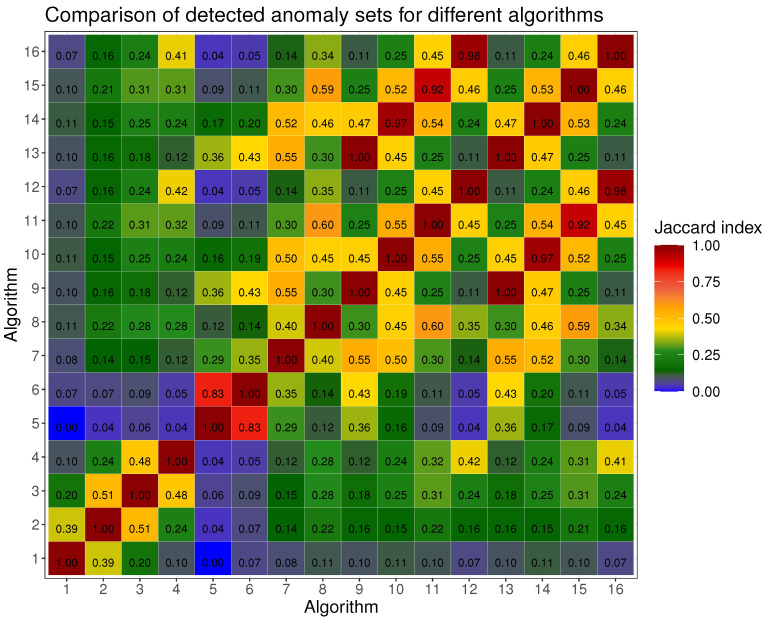
Comparison of results of potential anomalies detection with (4) evaluated with Jaccard index (8). Types of preprocessing and values of ϵ are in Table 3. The *k* parameter in (4) was arbitrarily set to 3.

**Figure 2:**
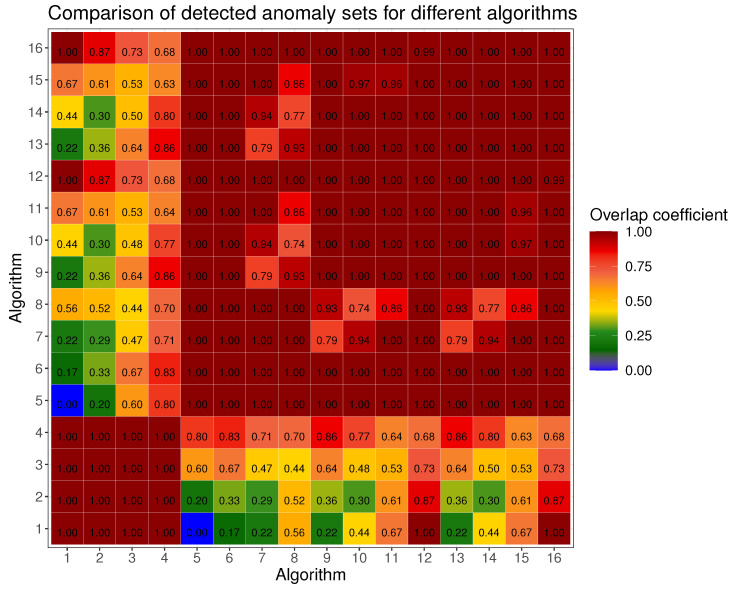
Comparison of results of potential anomalies detection with (4) evaluated with Overlap coefficient (9). Types of preprocessing and values of ϵ are in Table 3. The *k* parameter in (4) was arbitrarily set to 3.

**Figure 3:**
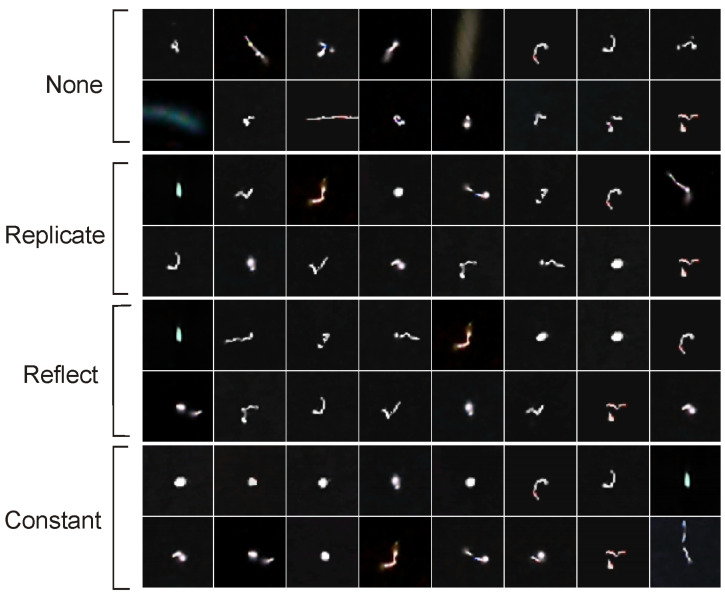
The first sixteen potential anomalies for each of the four preprocessing methods calculated for basic PCA with (α=2.4,k=3) in (4).

Figure 1 and Figure 2 present comparison of results of potential anomalies detection with (4) evaluated with (8) and (9). Types of preprocessing and values of ϵ are in Table 3. The *k* parameter in (4) was arbitrarily set to 3. The first sixteen potential anomalies for each of the four preprocessing methods calculated for basic PCA with (α=2.4,k=3) are shown in Figure 3.

**Figure 4:**
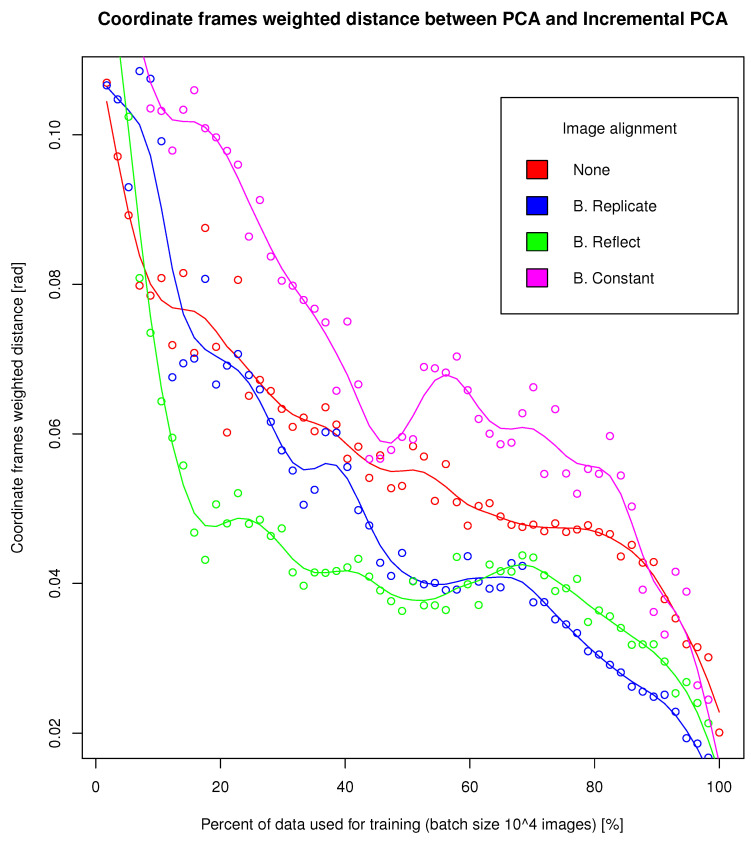
Comparison of coordinate frames obtained with basic PCA to coordinate frames obtained with Incremental PCA for a different number of data used when approximating PCA with Algorithm 2. Each point on the plot shows the cfd value (7) for the PCA coordinate axes calculated on the whole data and the coordinate axes calculated by Incremental PCA on a certain percentage of the whole data, that is, for example, the PCA axes calculated on the whole set with B. Replicate preprocessing and the axes calculated with Incremental PCA with B. Replicate preprocessing calculated on 10%, 20%, 30% of the data etc.

The next stage of the evaluation was to test the effectiveness of using Incremental PCA (see Section 2.6) in the procedure for detection of potential anomalies in dateset under condition of continuously incoming data (see Section 2.7). In order to do so, we made a comparison of coordinate frames obtained with basic PCA to coordinate frames obtained with Incremental PCA for a different number of data used when approximating PCA with Algorithm 2. The results are shown in Figure 4. Each point on the plot shows the cfd value (7) for the PCA coordinate axes calculated on the whole data and the coordinate axes calculated by Incremental PCA on a certain percentage of the whole data, that is, for example, the PCA axes calculated on the whole set with B. Replicate preprocessing and the axes calculated with Incremental PCA with B. Replicate preprocessing calculated on 10%, 20%, 30% of the data etc. We presented the selected cfd values for this evaluation in Table 4. For Incremental PCA, we assumed a batch size (bs) of 10,000.

**Figure 5:**
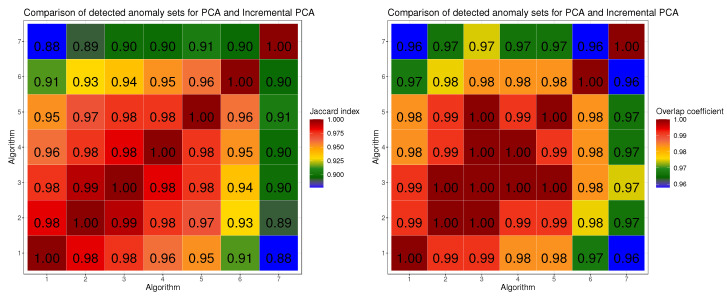
A comparison of Jaccard Index and Overlap Coefficient for the method of finding potential anomalies (4) with the parameters (α=2.4,k=3) for PCA and Incremental PCA counted on increasing numbers of data. We performed embedding and potential anomalies detection on the entire dataset.

**Figure 6:**
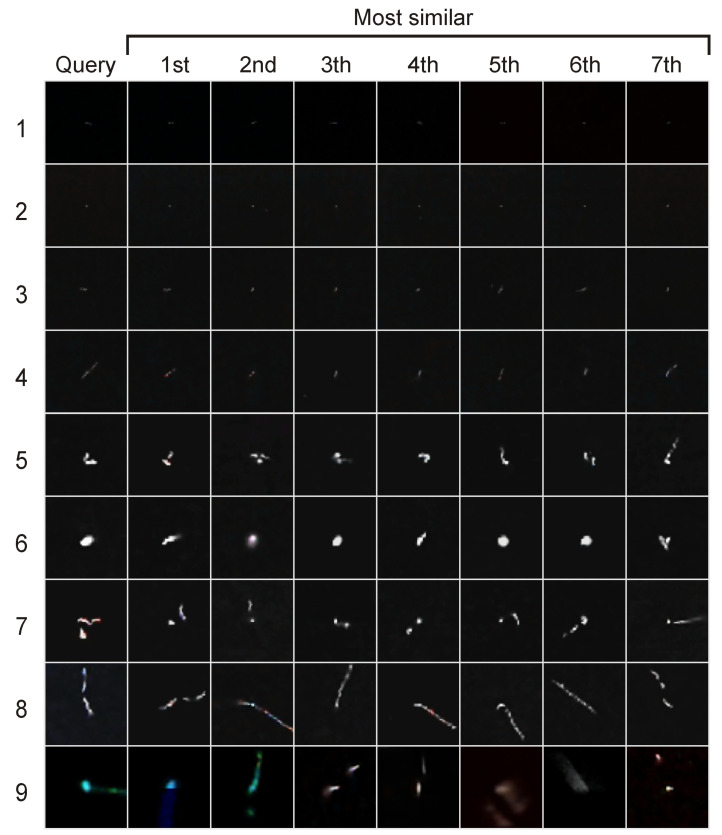
Test of the effectiveness of the method that detects similar objects according to Equation (6). For this purpose, we used the preprocessing algorithm B. Reflect and we generated features using basic PCA. The first column contains image $I_{j}$ (see Equation (6)). Each subsequent column contain the most similar images, the further to the left the Euclidean distance between embedding $E_{i}$ and $E_{j}$ is higher (second from left is most similar to first, first from right is the least similar from all seven).

Then we made a comparison of Jaccard Index and Overlap Coefficient of the method for finding potential anomalies (4) with the parameters (α=2.4,k=3) for PCA and Incremental PCA calculated on increasing numbers of data. Since the results for each image alignment were very similar on Figure 5 we present the results for B. Reflect only. We always performed embedding on the entire dataset and we calculated Incremental PCA for some subset of the data, thus simulating a constant increment of the data on which embedding is performed relative to the data used when counting embedding. The number of data used by Incremental PCA is coded in Figure 5 as follows:

1. basic PCA (calculated on full dataset),
2. Incremental PCA calculated on $56\cdot 10^{4}$ images.
3. Incremental PCA calculated on $46\cdot 10^{4}$ images,
4. Incremental PCA calculated on $36\cdot 10^{4}$ images,
5. Incremental PCA calculated on $26\cdot 10^{4}$ images,
6. Incremental PCA calculated on $16\cdot 10^{4}$ images,
7. Incremental PCA calculated on $6\cdot 10^{4}$ images,

The last step of the evaluation is to check the effectiveness of the method that detects similar objects according to Equation (6). For this purpose, we used the preprocessing algorithm B. Reflect and we generated features using basic PCA. We do not present the results obtained with Incremental PCA because, as will be shown in the discussion, they are virtually identical to basic PCA. We selected 9 sample images representing characteristic shape morphologies of particle tracks in the dataset and found k=7 most similar images according to Equation (6). We presented the results in Figure 6.

## 4. Discussion

Based on the results shown in Table 2, it can be concluded that the individual coordinate frames calculated on datasets with different embeddings differ from each other considering the cfd measure (7). In the case of lack of preprocessing (None) and B. Constant the differences between the obtained coordinate frames are the largest. This is due to the fact that B. Constant attaches black pixels on borders to the resulting image, which are not present in such numbers in raw images. There is a little difference between coordinate frames calculated on data processed with B. Reflect and B. Replicate, it amounts 0.042 rad. Although the difference is small, there is no guarantee that the embedding calculated with PCA on the set preprocessed with one method can be used interchangeably with the embedding calculated on the set preprocessed with another method. The choice of a particular preprocessing method determines the necessity of its use in subsequent stages of dataset analysis.

Although the different methods create different embedding of the images, the sets of anomalies they find are not significantly different. According to the results in Figure 1 and Figure 2, the number of anomalies found naturally decreases as ϵ increases. This can be observed when comparing two anomalies detection methods with a larger and smaller ϵ value–the Jaccard Index has a smaller value when there is a larger difference of ϵ between those two methods. As expected for a certain preprocessing method, as the ϵ decreases, new objects are added to the set of anomalies without removing those found with a larger ϵ. This can be observed from the Overlap Coefficient, which always has a value of 1 within a single preprocessing method regardless of ϵ. It can also be seen from the Overlap Coefficient analysis that the use of a preprocessing method (other than None) results in each of the detection algorithms returning a very similar set of potential anomalies–OC equals almost always 1 and the smallest value is in the case of B. Constant ϵ=2.4 and B. Replicate ϵ=2.8 and equals 0.74. If we compare embedding based on preprocessing None with other methods Overlap Coefficient ranges from 0 to 1, which means that different sets of potential anomalies are returned. Thus, one can conclude that preprocessing affects the anomalies that we detect. In the case of the Jaccard Index, the values of this coefficient are in most cases less than 1. This means that for the same values of ϵ, the different preprocessing methods affect embedding in such a way that they search for sets of potential anomalies of different quantity. This confirms the results from Table 2 that the coordinate frames are different from each other and the distances between objects in the PCA-designated spaces are also different.

When designing potential anomaly search algorithm using PCA embedding, we do not define the particle trajectory morphologies of interest. We expect that if we do not apply preprocessing but work on embedding generated from raw dataset (in our case preprocessing equals to None), the returned potential anomalies are different from those found when we apply preprocessing. This expectation is confirmed in Figure 3. The set of the first 16 retrieved anomalies for each method at ϵ=2.8 in the case of preprocessing None returns a dataset different from those returned by Replicate, Reflect and Constant. However those three preprocessing methods returns very similar particle tracks. This means that moving and rotating the objects so that the largest variance of bright points is along the horizontal axis significantly affects the result. Basing on what we know about PCA in other image domains, it can be concluded that image alignment is a beneficial process for variance analysis. For this reason, we recommend the use image aligning. The sets of potential anomalies returned by proposed algorithm do not contain any typical morphologies of particle tracks shapes (see, for example, the results of Figure 3). Thus, one can conclude that our proposed method effectively filter-off typical (in terms of analysis of variance) shapes of particle tracks by searching for those that can be treated as significantly different from the others in the dataset.

Based on the results from Table 4 shown in Figure 4, it can be seen that as the number of data processed by Incremental PCA increases (in our case with batch size set to 10,000), the cfd between PCA approximation and basic PCA expressed in radians decreases. Already for an approximately 40% of dataset, the difference between those two values is between 0.04 and 0.06 radians. The similarity of the coordinate frame calculated with basic PCA and Incremental PCA also affects the similarity of the obtained embedding and thus the detected anomalies. We performed such an analysis for preprocessing B. Reflect. As can be seen in Figure 5 for Incremental PCA calculated on 97% of data with batch size 10,000, J=98, OC=0.99, so the returned sets of potential anomalies are almost identical. As the data used to calculate Incremental PCA decreases, both coefficients also decrease but not significantly. For Incremental PCA calculated on 80% of data J=98, OC=0.99, for 62% J=96, OC=0.98, for 45% J=95, OC=0.98, for 28% J=91, OC=0.97, for 10% J=0.88, OC=0.96. This means that using Incremental PCA, which is recalculated with incoming data with a batch size of 10,000, we get almost identical anomalies detection results as for basic PCA. It can be concluded that our method, if the dataset is shuffled (and is representative) a small portion of the dataset used to calculate Incremental PCA can detect almost identical set of anomalies as calculated with basic PCA. The method we proposed in Section 2.7 for detecting of potential anomalies in large dataset under condition of continuously incoming objects works almost identically to the method using the entire dataset for PCA calculation. As a result, the approach we have proposed significantly reduces the memory and computational requirements of the algorithm for detecting anomalies and makes it possible to use it for big datasets.

Also the use of (6) to detect similar objects works as expected. It returns morphologically similar objects to the one being searched for. The results shown in Figure 6 for B. Reflect confirm that the returned objects $I_{i}$ have a very similar shape to the searched image $I_{j}$. Thanks to using image aligning, the method is not sensitive to translation and rotation of objects in images. As can be seen, the method based on (6) handles well the morphology of dots, lines, worms and various types of complex shapes. When searching for similar objects, the method also returns objects with similar levels of background noise, which may not be entirely beneficial (compare first and second row in the Figure 6). At the moment, however, with the preprocessing method described by Algorithm 1 it is not possible to remove the background. This is a certain drawback of that method if it will be applied to search for morphologically similar objects not considering background. Despite this fact, its search results give very satisfactory results in terms of morphology search.

The proposed anomalies detection algorithm worked as expected. The images it found have anomalous features according to their definition (4), that is, they contain traces of potential particles whose morphology differs significantly from typical image classes, that is, dots, lines and worms. The use of PCA as a feature extraction method did not create concentric clusters of objects. Due to this fact, one cannot use distance-based measures to find the central object of potential clusters, e.g., the “most typical dot class trajectory” around which there are similar objects. This behavior was expected because PCA does not statistically differentiate the correct signal from background noise present in some images. For this reason, density-based clustering seems to be an appropriate approach for grouping objects with similar morphology. Because (4) defines anomalies using a density-based approach, it is impossible to say which potential particle trace is “more anomalous” than the other. By controlling the parameters (α,k) in (4) and using various types of preprocessing, we have the ability to search the entire dataset. As we indicated in Figure 1 and Figure 2, the preprocessing method slightly affects the returned sets of anomalies.

We cannot exclude the possibility that some of these images are artifacts due to the access of visible light to the CMOS array. At this stage, we do not yet know the physical interpretations of the anomalies we are detecting. The main goal of our study was to create a method that would allow us to find them efficiently in large data sets. The physical interpretation of the results obtained is beyond the scope of this work and requires further research. Our proposed method is intended to be a useful mathematical tool for defining and finding potential anomalies.

**Figure 7:**
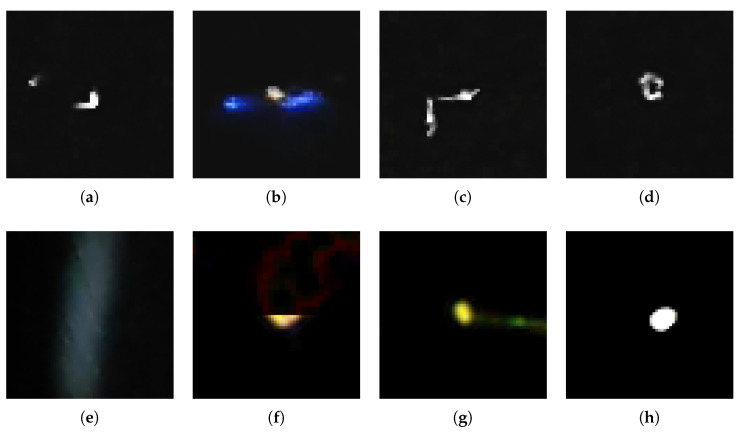
Examples of anomalies detected by the proposed method with parameters (α=2.3,k=5), B. Replicate preprocessing, basic PCA. We chose them because they represent a variety of deviations from the typical shapes of expected most typical trajectories: (**a**) two separated signals, (**b**) energy deposit wit colored halo effect, (**c**) worm-like signal with unexpected right angle, (**d**) untypical closed loop trajectory, (**e**) wide band with low energy deposit, (**f**) probably corrupted image file, (**g**) colored energy deposit with tail, (**h**) dot-like signal with too large energy deposit.

In Figure 7 we present examples of anomalies detected by the proposed method with parameters (α=2.3,k=5), B. Replicate preprocessing, basic PCA. We chose them because they represent a variety of deviations from the typical shapes of expected most typical trajectories. Figure 7a contains a clearly separated trajectories similar in shape to a dot and a worm. Figure 7b contains a circular shape in the center (larger than a typical dot) and there is a halo surrounding it, which affects CMOS sensor less than the core of the potential hit. Figure 7c looks like a typical worm, but the angle between its two parts is close to a right angle, which is unusual. Figure 7d also morphologically resembles a worm, however the trajectory forms a closed loop. Figure 7e contains a relatively wide rectilinear band, probably with a low energy deposit, which resembles a cloud. Figure 7f is probably the result of image file corruption because it looks like it consists of two images separated horizontally. Figure 7h contains a single circular area, but much larger than typical dot class representatives. In contrast, Figure 7g contains a large dot having an additional linear tail.

## 5. Conclusions

In conclusion, the method proposed in this paper for detecting potential anomalous cosmic ray particle tracks in big data image dataset acquired by CMOS proved to be effective in terms of the returned results. The use of Incremental PCA allowed approximation of *V* matrix which might be updated at runtime. Incremental PCA results with almost identical embedding as basic PCA. This significantly reduces the necessary calculations in terms of memory complexity so that our method can be used for big data. The use of intuitive parameters of the potential anomalies detection algorithm based on object density in embedding space makes our method intuitive to use. By manipulating the pair (ϵ,k) in (4), we can explore outliers and calibrate the algorithm for our needs with polynomial computational complexity even if we do not use parallel computing. The proposed method (6) can also be used to find similar objects, which gives it the potential, for example, to be used in minimal distance-based classification and image database querying. This application is worth further investigation as it would allow interactive exploration of the whole CREDO experiment dataset in real time, which is an important issue in terms of science and cognition.

## Figures and Tables

**Table 1:** Image aligning methods we used during dataset preprocessing. Column titled “Aligning and pixel extrapolation with example” gives example results of pixel extrapolation algorithm for borders.

Image Alignment	Aligning and Pixel Extrapolation with Example
None	Algorithm 1 is not applied (further processing of rough data)
B. Constant	Algorithm 1, ooo|abcd|ooo with specified o
B. Reflect	Algorithm 1, dcb|abcd|cba
B. Replicate	Algorithm 1, aaa|abcd|ddd

**Table 2:** A comparison of the resulting coordinate frames computed using basic PCA for four different preprocessing algorithms. The comparison of coordinate systems is done using coordinate frames weighted distance (cfd) and it is measured in radians.

	None	B. Constant	B. Reflect	B. Replicate
None	0	0.531	0.176	0.178
B. Constant	0.531	0	0.520	0.522
B. Reflect	0.176	0.520	0	0.042
B. Replicate	0.178	0.522	0.042	0

**Table 3:** Description of Algorithms in Figure 1 and Figure 2. Columns show algorithm id, type of preprocessing and value of ϵ in (4).

Algorithm id	Image Alignment	α
1	None	3.0
2	None	2.8
3	None	2.6
4	None	2.4
5	B. Constant	3.0
6	B. Constant	2.8
7	B. Constant	2.6
8	B. Constant	2.4
9	B. Replicate	3.0
10	B. Replicate	2.8
11	B. Replicate	2.6
12	B. Replicate	2.4
13	B. Reflect	3.0
14	B. Reflect	2.8
15	B. Reflect	2.6
16	B. Reflect	2.4

**Table 4:** Coordinate frames weighted distance between PCA and Incremental PCA. Weighted distance is expressed in radians [rad]. Each batch contains $10^{4}$ images.

Batch Number	None	B. Constant	B. Reflect	B. Replicate
1	0.107	0.107	0.115	0.133
3	0.089	0.093	0.102	0.117
5	0.078	0.107	0.074	0.104
7	0.072	0.068	0.059	0.098
9	0.071	0.070	0.047	0.106
11	0.072	0.067	0.051	0.100
13	0.081	0.071	0.052	0.096
15	0.067	0.066	0.049	0.091
17	0.063	0.058	0.047	0.080
19	0.062	0.051	0.040	0.078
21	0.064	0.060	0.041	0.075
23	0.057	0.056	0.042	0.075
25	0.054	0.048	0.041	0.057
27	0.053	0.041	0.038	0.058
29	0.058	0.040	0.040	0.059
31	0.051	0.040	0.037	0.069
33	0.051	0.039	0.044	0.070
35	0.050	0.040	0.037	0.062
37	0.049	0.039	0.042	0.059
39	0.048	0.042	0.044	0.063
41	0.047	0.038	0.041	0.055
43	0.047	0.035	0.039	0.055
45	0.048	0.031	0.035	0.055
47	0.047	0.029	0.036	0.060
49	0.045	0.026	0.032	0.050
51	0.043	0.025	0.032	0.036
53	0.035	0.023	0.025	0.042
55	0.031	0.019	0.024	0.026
57	0.020	0.010	0.012	0.013

## Data Availability

Source codes can be downloaded from: https://github.com/browarsoftware/anomalies_bigdata accessed on 20 December 2023.
